# Pictorial essay: Orbital tuberculosis

**DOI:** 10.4103/0971-3026.59744

**Published:** 2010-02

**Authors:** Mahender K Narula, Vikas Chaudhary, Dhiraj Baruah, Manoj Kathuria, Rama Anand

**Affiliations:** Department of Radiodiagnosis, Lady Hardinge Medical College and Associated Smt. Sucheta Kriplani and Kalawati Hospitals, New Delhi - 110 001, India

**Keywords:** Computed tomography, lacrimal gland, orbital tuberculosis

## Abstract

Tuberculosis of the orbit is rare, even in places where tuberculosis is endemic. The disease may involve soft tissue, the lacrimal gland, or the periosteum or bones of the orbital wall. Intracranial extension, in the form of extradural abscess, and infratemporal fossa extension has been described. This pictorial essay illustrates the imaging findings of nine histopathologically confirmed cases of orbital tuberculosis. All these patients responded to antituberculous treatment.

## Introduction

Tuberculosis is a major cause of morbidity and mortality in the third-world countries.[1,2] Orbital tuberculosis is rare, even in endemic areas.[3] Malignancy, developmental anomalies, and nontuberculous infections are the common orbital lesions noted in children.[4] Orbital tuberculosis is relatively more common in children, girls more likely to be affected than boys.[5] The disease is usually unilateral and slowly progressive. It has an insidious onset, with patients reporting that symptoms had been present for months to years. The left orbit is more commonly involved than the right.[5]

## Discussion

The primary tuberculous focus is commonly pulmonary, but extrapulmonary sites, such as cervical lymphadenopathy or abdominal disease, may be present.[5] Hematogenous spread from a primary tubercular focus or contiguous spread from paranasal sinuses may affect the orbit.[6] Orbital tuberculosis usually presents with destruction of bone (most commonly the frontal and sphenoid bones), with or without sclerosis, extraconal inflammation/abscess formation, extension into the infratemporal fossa, or intracranial (usually extradural) extension. Lacrimal gland involvement (enlargement/abscess) is also a common presentation. Involvement of the lateral wall suggests a hematogenous source of infection.[6] Involvement of the medial wall of the orbit is suggestive of spread of infection from an adjacent paranasal sinus. Orbital tuberculosis may present only with ocular lesions.[7]

The radiological features of orbital tuberculosis have been reported in only a few studies. Here, we describe the imaging features of nine cases of orbital tuberculosis (ages 1-15 years; seven boys and two girls) who presented with painless/painful orbital and/or lid swelling, proptosis, and ophthalmoplegia. In all the patients, routine laboratory investigations, chest radiographs, and orbital USG followed by CT scan were performed. The imaging findings were correlated with clinical and histopathological findings.

Although we did not use MRI as an imaging modality, the multiplanar capability and lack of bony artefacts makes MRI an excellent modality in the evaluation of orbital masses. It differentiates between different types of masses and also determines the extent of the lesion involving the lacrimal gland fossa and the brain. The use of fat suppression techniques combined with post contrast sequences significantly improves the visualization of subtle masses.[8]

### Bony involvement

**Figure 1 (a-d):**
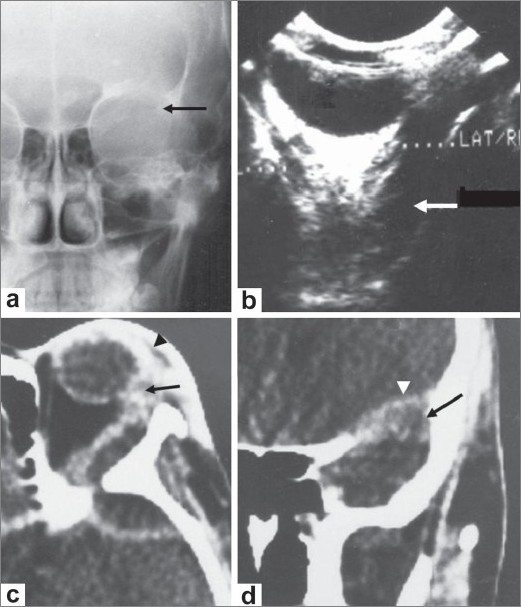
A 11-year-old girl presented with painless, nonpulsatile proptosis of the left eye of 3 months' duration. Caldwell view radiograph (a) shows destruction of the greater wing of the sphenoid on the left side (arrow). B-scan USG of the orbit (b) shows a hypoechoic collection in the extraconal space in the retroorbital region (arrow). Axial contrastenhanced CT scan (c) of the orbit, shows left-sided proptosis, lacrimal abscess (arrow), and preseptal thickening (arrowhead). Coronal, contrast-enhanced CT scan (d) shows destruction of the greater wing of the sphenoid (arrow) and intracranial extension (arrowhead).

**Figure 2 (a-d):**
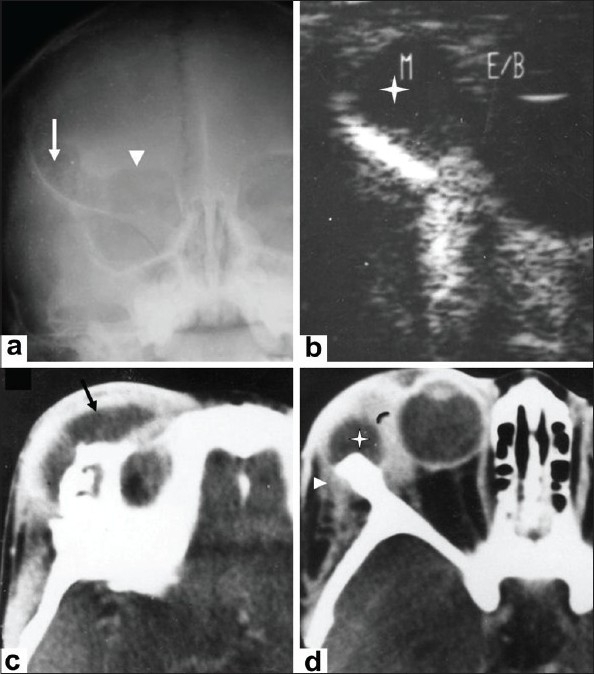
A 4-year-old boy presented with right-sided orbital swelling, with mild pain and restriction of eye movement. Caldwell view radiograph (a) shows an osteolytic lesion in the frontal bone (arrow) and destruction of the right orbital roof (arrowhead). B-scan USG of the orbit (b) shows a hypoechoic collection (asterisk) located superolaterally in the region of the lacrimal gland (lacrimal gland abscess). Axial contrast-enhanced CT scans (c,d) of the orbit, show an abscess in the lacrimal region, with rim enhancement (asterisk in d); the abscess is seen extending into the soft tissues superolaterally (causing preseptal thickening) (arrow in c) and into the infratemporal fossa (arrowhead in d).

**Figure 3 (a-c):**
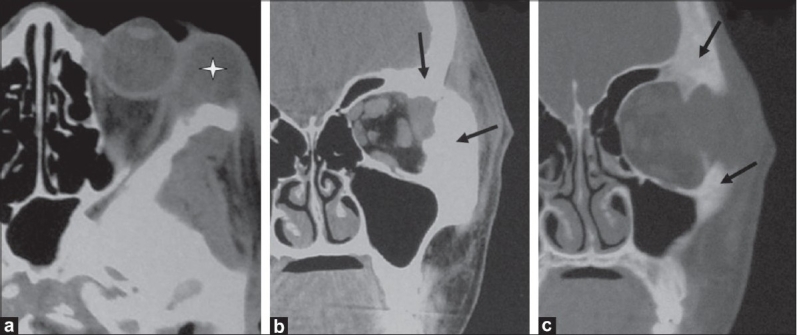
A 15-year-old boy presented with unilateral painless, nonpulsatile proptosis of the left eye. Axial contrast-enhanced CT scan (a) shows a left lacrimal abscess (asterisk). Coronal CT scans (b,c) show irregularity, destruction, thickening, and sclerosis of the adjacent frontal and zygomatic bones (arrow)

**Figure 4 (a-d):**
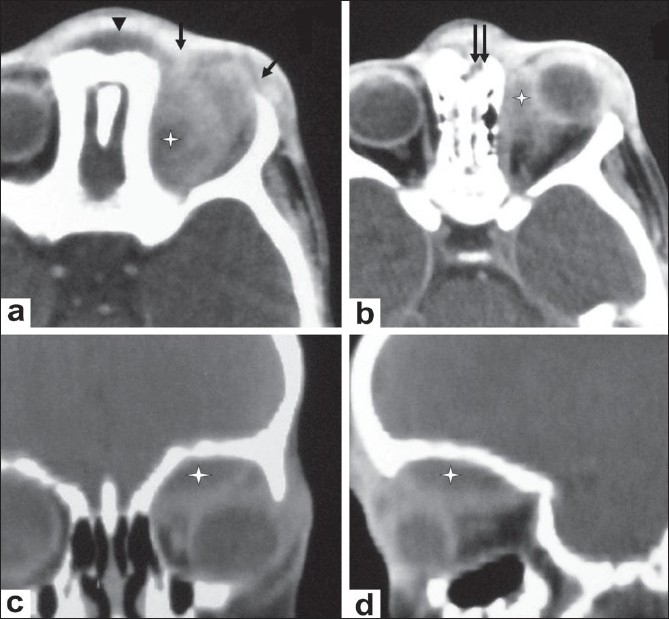
A 1-year-old child presented with mild pain and swelling of the left eye. Axial contrast-enhanced CT scans (a,b) show an extraconal orbital abscess (asterisk in a) with extension into the preseptal tissues (arrows in a) and into the soft tissue of the root of nose (arrowhead in a). There is involvement of adjacent ethmoid sinuses, with irregularity and destruction of their walls. Irregularity and destruction of the orbital plate of the frontal bone and nasion is also seen (double arrow in b). Coronal (c) and sagittal (d) CT scan reconstructions also demonstrate the extraconal orbital abscess (asterisk)

**Figure 5 (a-c):**
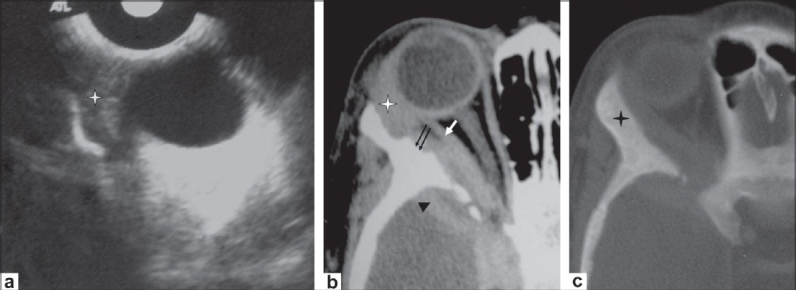
A 10-year-old boy presented with unilateral, painless, nonpulsatile proptosis of the right eye. B-scan USG of the orbit (a) shows a hypoechoic mass/thickening in the superolateral aspect of the orbit (enlarged lacrimal gland) (asterisk), with extension into the retro-orbital extraconal space. Axial contrast-enhanced CT scan (b) delineates the orbital inflammation with enlargement of the lacrimal gland (asterisk) and thickening of the lateral rectus muscle (arrow). Intracranial extension (extradural inflammatory mass with granulation tissue) (arrowhead) with involvement of the sphenoid bone (double arrow) is also seen. Axial CT scan (c), shows thickening and sclerosis of the involved frontal and sphenoid bones on right side (asterisk)

**Figure 6 (a-c):**
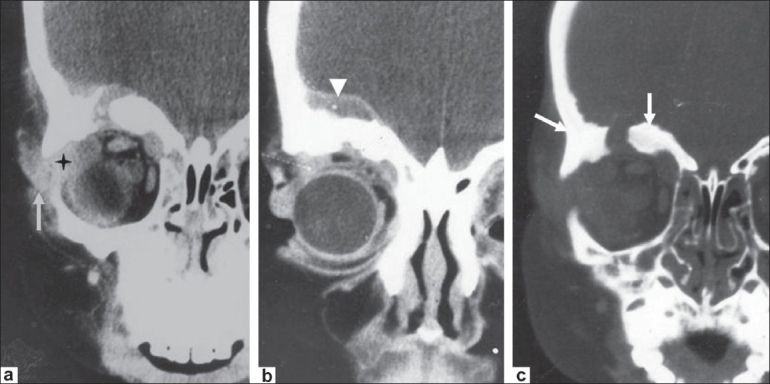
A 7-year-old boy presented with unilateral, painless, nonpulsatile proptosis of the right eye of 2 months' duration. Coronal contrast enhanced CT scans (a,b) of the orbit, show a hypodense mass in the lacrimal region (asterisk in a) with extension into the infratemporal fossa (arrow in a) and also intracranially (extradural abscess) (arrowhead in b). Coronal CT scan (c), shows associated destruction of the orbital plate of the frontal and zygomatic bones (arrows). The lateral rectus cannot be defined separately.

Orbital tuberculosis usually involves the bones of the orbital wall, viz. the orbital plate of the frontal bone, the sphenoid, or the zygomatic bone. Sphenoidal extension has been described but is rare.[5] Zygoma involvement associated with lower lid tuberculosis has also been reported.[9] Tuberculous periostitis is the usual manifestation of tuberculous infection and usually affects the outer margin of the orbit.[10] Bony involvement can also be seen in the form of cortical irregularity and destruction [Figures 1a, 1d, 2a, 2c, 3a, and 4b]. Bony thickening and sclerosis are seen in long-standing cases [Figures 3b, 3c, 5b, 5c, and 6a–c]. Other causes of orbital bone destruction in the pediatric age-group are neuroblastoma, which normally does not have an associated abscess and Ewing sarcoma, which usually shows a spiculated periosteal reaction with a soft tissue mass.

### Extraconal orbital inflammation/abscess

**Figure 7 (a-d):**
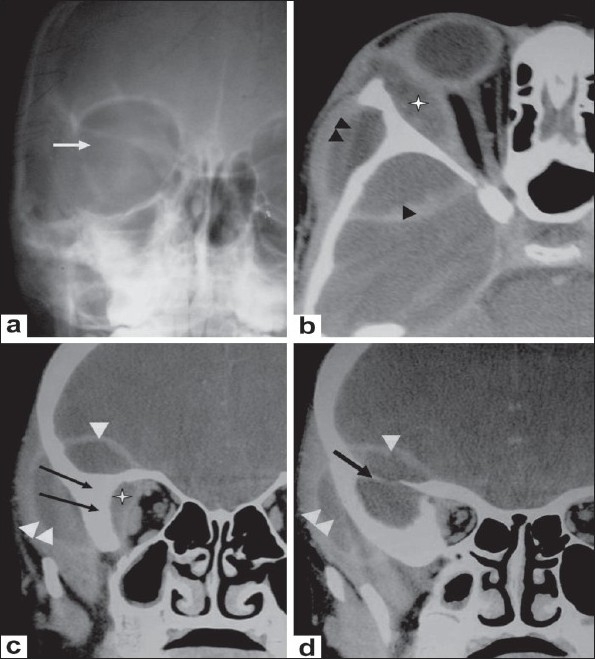
A 10-year-old boy presented with unilateral, painless, nonpulsatile proptosis of the right eye with low-grade fever. Caldwell view radiograph (a) shows loss of definition and fuzziness of the greater wing of the sphenoid on the right side (arrow). Axial (b) and coronal (c,d) contrast-enhanced CT scans of the orbit, show a rightsided extraconal and lacrimal abscess (asterisk in b,c), an intracranial extradural abscess (arrowhead), and an abscess in the infratemporal fossa region (double arrowhead). Irregularity and sclerosis of the sphenoid and zygomatic bones (arrow in c,d) is also seen.

**Figure 8 (a-d):**
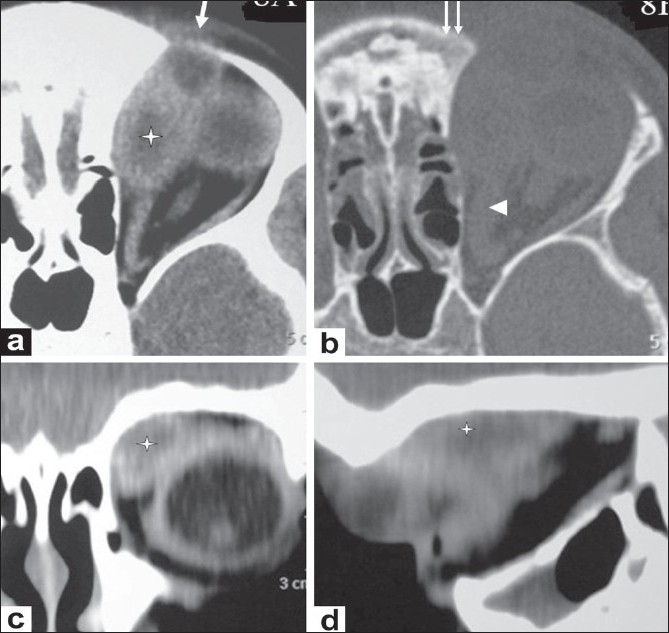
A 5-year-old boy presented with painless swelling (proptosis) of the left eye. Axial (a,b), coronal (c) and sagittal (d), CT scans show an extraconal orbital abscess (asterisk in a,c,d) in the superomedial region of the left orbit with extension into the preseptal tissue (arrow in a). The adjacent medial rectus muscle appears slightly thickened (arrowhead in b). The adjacent bone shows scalloping, and there is a focal area of subtle loss of definition of the cortical margin anteromedially (double arrow in b).

Orbital abscess/inflammation is usually seen in the extraconal compartment [Figures 1b, 7a–c, 5a, 5b, 4a–d, and 8a–d].

### Intracranial extension

Coexistence of ocular and central nervous system tuberculosis is known,[11] but orbital tuberculosis extending into the cranium has also been reported.[3,12,13] Intracranial extension is usually seen in the form of extradural abscesses [Figures 1d, 7b–d, 5b, and 6b].

### Infratemporal fossa extension

Extension of orbital tuberculosis into the infratemporal fossa has also been described.[3] [Figures 7b–d, 2c, 2d, and 6a].

### Lacrimal gland involvement

**Figure 9 (a,b):**
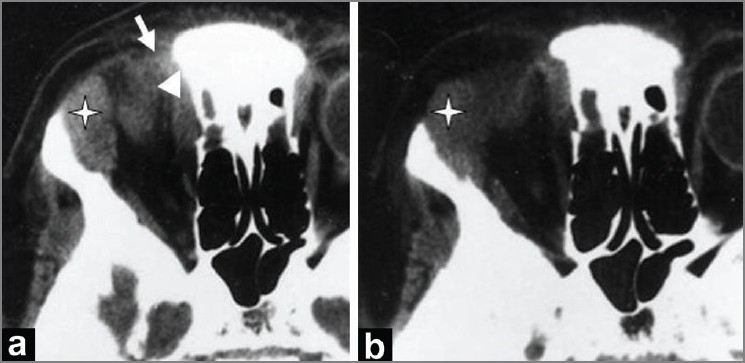
A 9-year-old girl presented with unilateral painless nonpulsatile proptosis of the right eye. Axial contrast-enhanced CT scans (a,b) of the orbit, show an enlarged lacrimal gland (asterisk), fine-needle aspiration cytology of which demonstrated tuberculous granulomas with caseation. Sight heterogenous soft tissue thickening (arrow in a) is also seen in the superomedial aspect of the orbit with slight irregularity of the adjacent bone (arrowhead in a)

Isolated involvement of the lacrimal gland has been described.[14] It may be seen either in the form of lacrimal gland enlargement [Figures 5c, 9a, and 9b] or abscesses [Figures 1c, 7b, 2b, 2d, and 3a]. Other causes of lacrimal gland enlargement include lymphoma and sarcoid; however, these conditions are usually bilateral and do not show orbital abscess or bony destruction.

### Preseptal thickening

The inflammation may extend to the preseptal tissues causing thickening [Figures 1c, 7b, 2d, 5b, 4a, and 8a].

## Conclusion

Tuberculosis of the orbit is rare. It is usually seen in the pediatric age-group. The disease is usually unilateral. The common presentations are proptosis, nontender or mildly painful orbital/lid swelling, and sinus formation. The predominant imaging features of orbital tuberculosis are involvement of the orbital bony wall and lacrimal gland, with soft tissue inflammatory mass/abscess formation. Intracranial and infratemporal extension is not uncommon. Patients presenting with isolated preseptal thickening need to be thoroughly investigated for presence of local disease and any underlying systemic focus. Although malignancy, developmental anomalies, and nontuberculous infections are much commoner causes of proptosis in childhood, tuberculosis should always be considered in the differential diagnosis of orbital masses. The clinician should have a high index of suspicion as orbital tuberculosis has probably been underdiagnosed in the past.
